# Food Quality Improvement of Soy Milk Made from Short-Time Germinated Soybeans

**DOI:** 10.3390/foods2020198

**Published:** 2013-05-21

**Authors:** Susu Jiang, Weixi Cai, Baojun Xu

**Affiliations:** 1 School of Biological Sciences, The University of Hong Kong, Hong Kong; E-Mail: h1292055@connect.hku.hk; 2 Food Science and Technology Program, Beijing Normal University-Hong Kong Baptist University United International College, Zhuhai, Guangdong 519085, China; E-Mail: weixicai@uic.edu.hk

**Keywords:** germination, physicochemical property, phytic acid, soy milk, total phenolic content, trypsin inhibitor activity

## Abstract

The objectives of this study were to develop soy milk with improved food quality and to enhance the functional attributes by incorporating short-time germination into the processing. Changes in trypsin inhibitor activity (TIA), phytic acid content and total phenolic content (TPC) in soy milk produced from soybeans germinated within 72 h were investigated to determine the optimum germination condition. Results from the present research showed significant (*p* < 0.05) improvement of TPC in cooked germinated soybean milk, while both the TIA and phytic acid content were decreased significantly (*p* < 0.05). In the subsequent evaluation on the quality attributes under the optimum germination condition, soy milk made from 28 h-germinated soybeans presented enhanced nutritional value and comparable physicochemical properties to conventional soy milk. The current approach provides a feasible and convenient way for soy-based product innovation in both household and industrial settings.

## 1. Introduction

Soy milk, the water extract of soybeans, is typically produced by grinding the soaked soybeans with water. As an inexpensive and convenient source of high quality proteins, soy milk is one of the most important traditional beverages that are consumed widely in Asian countries, including China, Japan, Korea, Singapore and Thailand. In recent decades, extensive evidence has indicated the strong relationships between soy food consumption and health-promoting effects. Soy milk possesses a balanced nutrient combination, which is similar to cow’s milk, but free of cholesterol, gluten and lactose, plus favorable phytochemical compounds linked to health. Consequently, it has drastically spread its popularity to Western consumers in European countries, Australia and the United States, especially among vegetarians, milk allergy patients or people of lactose-intolerance, who use soy milk as a dairy alternative. In response to a gradual increase in sales and consumption, various new products have been introduced into the soy milk market. Some basic changes are made to the flavor or the soybean source. However, the most recent innovations are focusing on producing “functional soy milk”.

Functional soy milk can be considered as soy milk that contains extra bioactive components and may help to enhance health or lower risk of diseases. Soybean is a good source of phenolic compounds with antioxidant properties and has an extraordinarily high amount of isoflavones, a group of phytoestrogens that have been reported to possibly lower the risk of hormonal and age-related diseases. However, the presence of natural anti-nutrients, such as trypsin inhibitors (TI), lectins, phytic acids and indigestible oligosaccharides, has limited its consumption. Thus, modifying the processing methods could be an effective way to improve the health-promoting bioactive components and/or reduce the undesired compounds originally present in soybeans, to support functional soy milk product development.

The heating process during conventional soy milk making considerably destroys most of the anti-nutritional factors in soy milk and improves the digestibility of soy protein, as well. However, compounds, like phytic acid, which interferes with the availability of calcium, is not reduced effectively [1]. At the same time, over-heating to eliminate trypsin inhibitor activity to a great extent can cause damage to amino acids, as well as loss in the overall nutritional value of soy milk. Lately, incorporating the fermentation process into soy milk production has become a popular method to improve the acceptability and health properties of soy milk. Research has shown that soy milk fermented with properly selected microbial species had major advantages in decreasing indigestible oligosaccharides, like raffinose and stachyose, and beany flavor (undesirable for most Western consumers). However, with respect to other anti-nutritional factors, such as trypsin inhibitors, no improvements were observed.

In this study, a change was made to the conventional soy milk making method by starting the process with short-time germinated soybeans in an attempt to overcome some of these limitations with soy milk. The germination process is a period of intense metabolic activity, incorporating processes, such as respiration, subcellular structural changes, macromolecular syntheses and hydrolysis, and conversion of stored proteins, fats and carbohydrates to support the initial seedling establishment [2]. It has long been the leading way to improve the nutritional value of soybeans, so as to increase its use. Although additional labor, work and cost are required to produce sprouted soybeans compared to the original seeds, germination is still recognized as an inexpensive and simple method, considering its multi-effects in enhancing nutritional value and lowering anti-nutritional factors. These include: (1) increases of vitamin contents (e.g., vitamin C and riboflavin) and mineral availability (e.g., calcium) [3]; (2) improvement of protein digestibility [4]; (3) hydrolysis of flatulent-causing oligosaccharides; (4) reducing levels of trypsin inhibitors, lectin, phytic acid and lipoxygenase activity, which lead to undesirable beany flavor [5,6]; and (5) enrichment of phenolics, isoflavone aglycones and saponin glycosides [4,6]. Many of these are closely related to soy milk quality and can be key attributes of soy milk. Thus, germination can be a very potent and efficient step for producing functional soy milk, as it covers the major benefits produced from other modifications, like fermentation, but possesses even more advantageous characteristics.

However, the effects of short-time germination on bioactive components are not very clear and may vary greatly with different germination conditions, and whether the patterns will change after conventional soy milk making process is still unknown. The current project aimed to establish optimum soybean germination conditions, under which the sprouted soybeans could be used to produce functional soy milk. The changes in TIA, phytic acid content and TPC in soy milk produced with soybeans germinated for different times were first investigated. The optimum germination condition with specific germination time was selected by comparing the values of the three indicators. In addition, a profile evaluation on the quality attributes was done to soy milk made from soybeans germinated at optimized conditions. Ideally, improvement was observed simultaneously in all three variables studied and further analyses helped to assess the overall food quality of soy milk. The data provided in this project would be valuable for the establishment of a novel soy milk line with functional properties and improved food quality compared to the conventional one. 

## 2. Materials and Methods

### 2.1. Soybean Materials

Yellow soybeans (*Glycine max* (L.) Merr., a small-seeded variety) cultivated in Hunan Province of China (harvested in 2010) were purchased from a local grocery in Zhuhai, China. A few seeds with defects were removed from samples.

### 2.2. Chemicals and Reagents

Trypsin from bovine pancreas and benzoyl-dl-arginine-*p*-nitroanilide (BAPA) were obtained from Sigma-Aldrich Co. (St. Louis, MO, U.S.A.). Phytic acid and Tris-base were purchased from Shanghai Yuanye Biotechnology Co., Ltd. (Shanghai, China). Folin-Ciocalteu reagent was supplied by Shanghai Sanjie Biotechnology Co., Ltd. (Shanghai, China). Sulfosalicylic acid and potassium sulfate were purchased from Tianjin Yongda Chemical Reagent Co., Ltd. (Tianjin, China). Iron (III) chloride hexahydrate was supplied by Sinopharm Chemical Reagent Co., Ltd. (Shanghai, China). The other chemicals were purchased from Guangzhou Chemical Reagent Company (Guangzhou, China) and Tianjin Damao Chemical Reagent Co., Ltd. (Tianjin, China). Unless otherwise stated, all the chemicals used were of analytical grade.

### 2.3. Germination of Soybean

Soybeans were cleaned and rinsed three times with tap water before being soaked overnight (~12 h) at room temperature with a water to dry bean ratio of 10:1, v/w. The soaked beans were drained, rinsed and placed in a semi-automatic germination machine (model JCD-001, Zhejiang Jianlong Technology, Yongkang, China), which watered the seeds every 15 min with tap water (~25 °C) automatically, and water was changed every 12 h manually. The germination process was carried out at room temperature (~25 °C) for 28 h, 50 h and 72 h, respectively.

### 2.4. Preparation of Soy Milk

For each batch of soy milk, 60 g of germinated soybeans or non-germinated soybeans underwent the same washing and soaking procedures (dry weight basis), were drained, rinsed and mixed with 540 mL tap water (water/dry bean = 9:1, v/w). To make raw soy milk, the mixture was subjected to a household blender (model QH-900B1, Joyoung Co., Ltd., Hangzhou, China) and blended for 5 min at low speed, while the cooked soy milk was prepared using a household soy milk maker (model JYD-F13P90, Joyoung Co., Ltd., Hangzhou, China). Both raw and cooked soy slurry was filtered through a mesh screen to obtain soy milk. The cooked one was immediately placed in an ice bath to cool down rapidly. For quality attribute analyses, liquid soy milk was sampled; the rest of the soy milk was sampled and freeze-dried by FreeZone Benchtop Freeze Dry System (Labconco Corporation, Kansas City, MO, USA) and stored at −20 °C for further determination of bioactive components.

### 2.5. Determination of Quality Attributes

#### 2.5.1. pH Value

The pH of soy milk was measured with a calibrated digital pH meter (model PHB-3, Shanghai Sanxin Meter Factory, Shanghai, China).

#### 2.5.2. Total Solids, Moisture and Ash

Total solids content was determined according to procedures described by Liu and Chang [7]. Approximately 10 g of soy milk was weighed into a pre-weighed crucible and placed in a hot air oven at 100 °C for 15 h. Dried residue was weighed after cooling in a desiccator to calculate total solids, and based on this, moisture content was also determined. Ash content was determined after burning in a muffle furnace at 550 °C for 12 h.

#### 2.5.3. Crude Protein, Fat and Carbohydrate

Crude protein and fat content were determined according to the method of China National Standardization Committee of Light Industry QB/T 2132-2008 [8]. Briefly, about 10 g of soy milk was sampled, respectively. Protein content was determined by the Kjeldahl method using a conversion factor of 6.25. Fat content was measured by weight after alkaline hydrolysis coupled with solvent extraction (ether and petroleum ether). Carbohydrate content was calculated by subtracting the moisture, protein, fat and ash content from the total mass.

#### 2.5.4. Viscosity

Viscosity was measured at 27 ± 1 °C using a Brookfield Viscometer (Model LV DV-II+Pro, Brookfield Engineering Labs., Inc., Middleboro, MA, U.S.A.) with spindle No.1 and a speed of 100 rpm.

### 2.6. Assay for Total Phenolic Content (TPC)

Phenolic compounds were extracted from freeze-dried soy milk powder using acidic acetone as the extraction solvent. Concisely, 0.5 g of dry powder was extracted twice with 5 mL of acidic acetone (acetone:water:acetic acid = 70:29.5:0.5, v/v/v) in dark, and the two extracts were combined for determination. The reason for selecting this solvent system was based on our previous study [9] that this solvent system gave the best yields of total phenolic contents and antioxidant activities. The TPC was determined based on a Folin-Ciocalteu assay using gallic acid (GA) as the external standard. The mixture solution prepared from sample extracts (50 μL), distilled water (3950 μL), Folin-Ciocalteu’s reagent (250 μL) and 7% Na_2_CO_3_ (750 μL) was vortexed and allowed to stand for 2 h. The absorbance was measured using a UV-visible spectrophotometer (TU-1901, Beijing Purkinje General Instrument Co., Ltd., Beijing, China) against a reagent blank at 765 nm. The results were calculated from the gallic acid calibration curve and expressed as milligrams of gallic acid equivalents per gram of freeze-dried soy milk (mg GAE/g).

### 2.7. Assay for Phytic Acid

Approximately 0.5 g of freeze-dried soy milk was defatted with 10 mL of petroleum ether. The residues were then extracted with 10 mL of 2.4% HCl by shaking on the orbit shaker for 14 h. After centrifugation, the supernatant was collected for analysis [10]. The phytic acid in the extract was determined according to a colorimetric assay described by Gao *et al.* [10], with slight modification. Exactly 0.1 mL of sample extract was diluted to 3 mL with distilled water and then thoroughly mixed with 1 mL of Wade Reagent (0.03% FeCl_3_·6H_2_O + 0.3% sulfosalicylic acid). The mixture was centrifuged at 5500 rpm for 10 min after standing for 30 min, and the absorbance of supernatant was immediately measured at 500 nm against distilled water. The phytic acid content was calculated from the calibration curve of phytic acid standard and expressed as milligrams of phytic acid (PA) per gram of freeze-dried soy milk (mg/g). 

### 2.8. Assay for Trypsin Inhibitor Activity (TIA)

The sample extract was prepared based on procedures of Hamerstrand *et al.* [11], with some modifications. Freeze-dried soy milk (0.5 g) was extracted with 50 mL of 0.01 N NaOH for 5 h, while being shaken constantly at room temperature. The suspension was then diluted to 100 mL with distilled water and allowed to stand for 2 h at 4 °C. The supernatant was collected and diluted, so that 2 mL of the extract could produce a trypsin inhibition rate between 40% and 60%. The TIA assay proposed by Hamerstrand *et al.* [11] was used, with some modifications. Briefly, to two test tubes for the sample and sample blank, 2 mL of diluted sample was added, while 2 mL of distilled water was added to another two tubes for the standard and standard blank. Trypsin standard solution (2 mL) was then added to sample tubes and standard tubes, and then all the tubes were vortexed and incubated in a water bath at 37 °C for 10 min. Then, 5 mL of BAPA solution preheated to 37 °C was added rapidly to each tube; the tubes were vortexed and incubated in a water bath at 37 °C for another 10 min before mixing with 1 mL of 30% acetic acid. After 2 mL of trypsin standard was added to tubes for standard and sample blanks, the mixtures in tubes were centrifuged at 6000 rpm for 10 min, and the supernatants were subjected to vacuum filtration. The absorbances of the standard and sample filtrates were measured at 410 nm against the respective blank. The TIA was expressed as trypsin inhibitor units (TIU) per gram of freeze-dried soy milk (TIU/g), as well as milligrams of trypsin inhibitor equivalent (mg TI/g) by dividing the TIU by 1900.

### 2.9. Statistical Analysis

All the assays were conducted at least in duplicate, and the results were expressed as the mean ± standard deviation. Analysis of variance (ANOVA) of the data was performed using the SPSS package (SPSS 17.0, SPSS Inc., Chicago, IL, USA) and Tukey’s HSD (Honestly Significant Difference) test, with a confidence interval of 95% used to determine significant differencesbetween means.

## 3. Results and Discussion

### 3.1. Effect of Germination on TPC in Soy Milk

Phenolics consist of a huge and diverse group of compounds, including flavonoids, phenolic acids and lignins, which are present in soy products in a relatively high amount. Their beneficial roles in human diet as bioactive compounds have been widely studied, with increasing evidence suggesting a role in reducing the risk of chronic diseases, such as cardiovascular diseases, diabetes, cancers, age-related eye problems and immune dysfunction, primarily attributed to the antioxidative property of phenolic compounds. Thus, phenolic compounds are the best candidates for developing value-added foods or nutraceuticals. The germination process is considered a potent way to increase phenolic content in plant seeds like soybeans, due to the biosynthesis and bioaccumulation of phenolic compounds as a defensive mechanism to survive under environmental stresses, like cold exposure [12].

**Figure 1:**
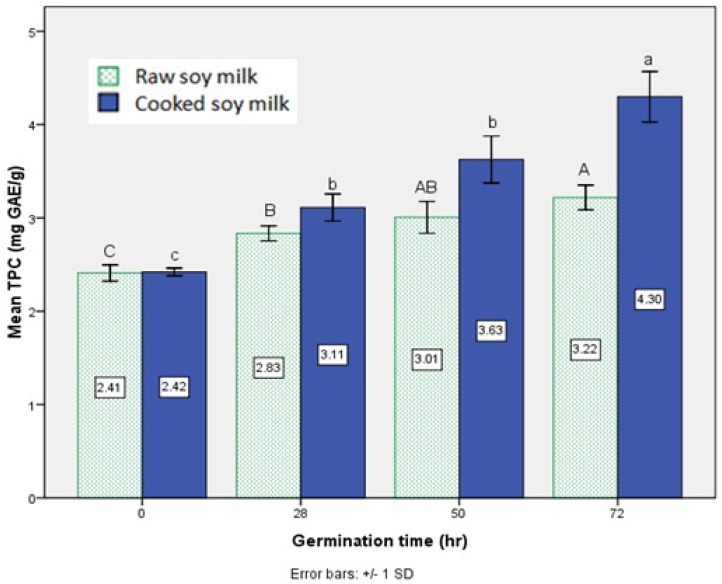
Effect of different germination time on total phenolic content (TPC) of soy milk. Bar data are expressed as the mean±standard deviation (*n*=3). Bars marked with the same capital letters indicate no significant (*p* < 0.05) differences among the raw soy milks. Bars marked with the same small case letters indicate no significant (*p* < 0.05) differences among the cooked soy milks.

Figure 1 shows the TPC values of soy milk made from soybeans germinated for 28 h, 50 h and 72 h, surprisingly, the germination process led to a continuous increase in TPC of both raw and cooked soy milk. While the maximum increase was observed for longer germination time (72 h), which exhibited a 78% increase in TPC value as compared to the value in non-germinated cooked soy milk, even germination for 28 h had a significant (*p* < 0.05) impact on TPC (29% increase). The raw soy milk group, due to a lack of the influence of thermal treatment, generated a comparable pattern of change to that observed in raw soybeans during germination by Lin and Lai [13]; at the same time, the cooked group tended to maintain the similar trend.

Thermal treatment carried out by the soy milk maker did not have a substantial effect on the final TPC value in the non-germinated soybean milk. This is contrary to data provided by Xu and Chang [14], which suggested that TPC significantly declined in soy milk after conventional thermal processing at 100 °C for 20 min. The discrepancy here could be attributed to the different processing equipment, as well as cooking manners used for preparing the raw and cooked soy milk. However, the results still implied that thermal treatment improved the TPC value of soy milk produced from the germinated 28 h, 50 h and 72 h soybeans for 9%–33.5% (Figure 1), possibly due to the release of the bound form phenolic acids or other simpler phenolics from the conjugated form. As indirect proof to the current results, Randhir and others [15] observed increases of phenolic content in plants, like soybean sprouts, after heat processing. As they summarized, the increase may come from the release of accumulated phenolics in cell vacuoles by heat or formation of new types of phenolics induced by the oxidation or polymerization reactions, which took place under heating conditions.

### 3.2. Effect of Germination on Phytic Acid Content

Phytic acid, a primary anti-nutritional factor, exists in soybean and its related products. In negatively charged salt form, it binds with ions of essential minerals, like calcium, iron and zinc, reducing their bioavailability or impairing the digestibility of proteins and carbohydrates by complexing with them. Although some recent research has interest in its antioxidant potential, to serve as an anti-tumor reagent for treating colon cancer [16], they only considered these at low intake doses and stressed the growth impairment effect for large consumption. Therefore, cutting down the phytic acid content in soy products, like soy milk, is favorable for any sake.

**Figure 2:**
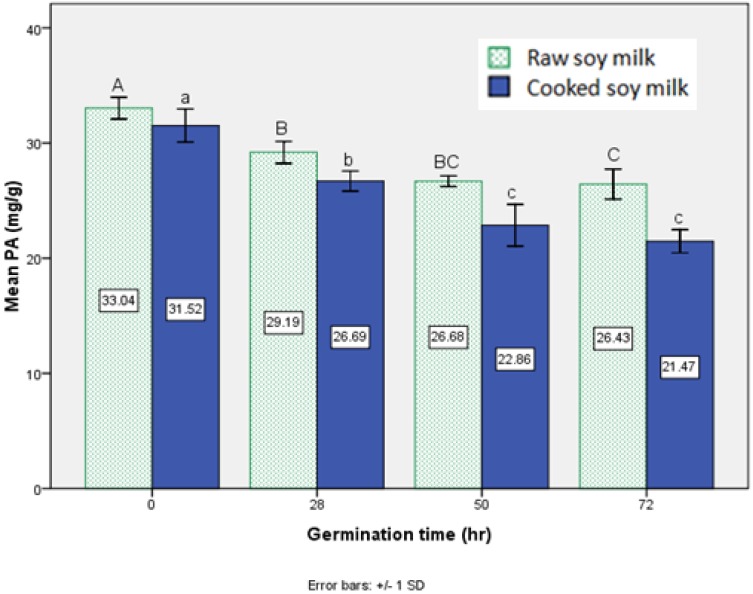
Effect of different germination time on phytic acid content of soy milk. Bar data are expressed as the mean±standard deviation (*n*=3). Bars marked with the same capital letters indicate no significant (*p* < 0.05) differences among the raw soy milks, and bars marked with the same small case letters indicate no significant (*p* < 0.05) differences among the cooked soy milks.

Figure 2 shows the phytic acid content in raw and cooked soy milk made from soybeans germinated for different times, according to which germination caused significant differences (*p* < 0.05) in the phytic acid content of soy milk produced from germinated soybeans or non-germinated soybeans, and longer germination time appeared to reduce the level substantially. It can be explained that soybeans take up phytate as an inorganic phosphate source during germination upon increased activity of intrinsic enzyme phytase [17]. Compared with the literature data, the tendency of reduction rates of phytic acid in the current study determined that for the raw soymilk made from soybeans germinated at the three time periods, they are similar to those determined by Rasha *et al.* [18] in germinated soybeans. Meanwhile, we noticed that the increased TPC negatively correlated with the decreased phytic acid content during germination. The mechanism behind this correlation remains unclear; it need to be further studied to figure out the reason. 

Although germination was more effective for decreasing the heat-stable phytic acid level as compared to thermal treatment, cooking also assisted in reducing phytic acid content, probably due to its hydrolysis into lower inositol polyphosphates or thermal degradation and interaction between phytic acid with protein or minerals to form insoluble complexes. In the current study, the combined effect of germination and thermal processing to produce soy milk decreased phytic acid content from 19% to 35%, higher than thermal treatment alone (5%), or only germinated for less than 72 h (19%). The synergistic effect on reducing phytic acid content is possibly due to phytic acid being more heat-stable in higher concentrations. The germination pre-treatment lowered phytic acid content in the raw soy milk; thus, more phytic acid degraded at the high temperature during cooking.

### 3.3. Effect of Germination on Trypsin Inhibitor Activity in Soy Milk

Probably the most notable anti-nutritional factors related to soy product are trypsin inhibitors. Soybeans contain two major types of trypsin inhibitors: the heat labile Kunitz soybean trypsin inhibitor (KSTI) and the heat stable Bowman-Birk inhibitor (BBI) specific to chymotrypsin [19]. Their inhibition effect on protease is linked to the loss of dietary protein digestibility, pancreatic hypertrophy or dysfunction and growth retardation, but may also be considered as a drug to reverse the propagation of some cancers. Nevertheless, the minimal TIA in soy milk is a warrant for the health of frequent consumers, especially children and vegetarians.

Table 1 shows that the TIA in raw and cooked soy milk was affected by different germination time. In the original raw soy milk, the concentration of trypsin inhibitor was 29.6 mg/g of freeze-dried soy milk powder. The thermal process carried out by the soy milk maker substantially decreased its content to 14.7% of the initial value; the reduction rate is similar to that of home cooking at 100 °C for 20 min and a little higher than that of the commercial ultrahigh-temperature processing (14.7%–24.1% residue) reported by Yuan *et al.* [20]. Although cooking was effective to eliminate the activity of heat labile KSTI [19], only part of BBI was inactivated during the process, which contributed to the residue amount of TIA. The effect on BBI was also compromised by the diluted liquid system of soy milk. It is widely accepted that reducing the TIA by up to 90% in soy milk provide the optimum nutritional value [21]. However, prolonged heating to reach this target generally results in the destruction of essential amino acids and other valuable components in soy milk [22].

**Table 1:** Effectof germination time on trypsin inhibitor (TI) activity of soy milk ^a^.

Germination time (h)	TIU/g		mg TI/g		Residue%	
	Raw	Cooked	Raw	Cooked	Raw	Cooked
0	56,245 ± 2741	8280 ± 485	29.60 ± 1.44 A	4.36 ± 0.26 a	100 ± 4.9	14.7 ± 0.9
28	40,552 ± 325	4685 ± 885	21.34 ± 0.17 B	2.47 ± 0.45 b	72.1 ± 0.6	8.3 ± 1.5
50	43,001 ± 2121	5397 ± 497	22.63 ± 1.12 B	2.84 ± 0.26 b	76.5 ± 3.8	9.6 ± 0.9
72	45,243 ± 1244	5894 ± 18	23.81 ± 0.65 B	3.10 ± 0.01 b	80.4 ± 2.2	10.5 ± 0.0

^a^ Results are the means of two replicates with standard deviation; Values marked with the same letter in the same column are not significantly different in mg TI/g (*p* < 0.05); TIU, trypsin inhibitor units.

When short-time germinated soybeans were used to make soy milk, the TIA dropped considerably to less than 10% after the same cooking procedure, and the maximum reduction of 91.7% was observed after 28 h of germination. The TIA decrease upon germination can be attributed to the hydrolysis of trypsin inhibitors to essential amino acids in favor of seedling growth during germination [23]. In soybeans, protease K1 plays a key role in initiating the degradation of both KSTI and BBI, whereas protease B1 and B2 are mainly responsible for the extensive proteolysis [24]. In addition, water was changed every 12 h during germination; leaching in the water may somehow contribute to the partial loss. Although germination significantly (*p* < 0.05) reduced the TIA value by about 20%–30% (in raw soy milk), the influence of germination time was not as conspicuous. It is interesting to note that the present result gives an indication that TIA decreased to some extent, but then slowly increased within 72 h of germination. The trend contradicts conclusions drawn by Kumar *et al.* [25] and Paucar-Menacho *et al.* [6], which suggested TIA decreased continuously with a soybean germination time up to 144 h, but our current results correspond well with Wilson’s research [26]. The new trypsin inhibitor form was observed in a study after about 48 h of germination of soybeans [25]. The altered active site of the new form increases the inhibitory activity [27] and may be the cause of the slight rise in TIA after 28 h. The differences in soybean materials and germination conditions are possible reasons for the discrete results of TIA during soybean germination from different studies. Still, the three selected germination times all led to improvement of soy milk quality in terms of TIA, without the application of excessive heat.

### 3.4. Selection of Optimum Germination Condition

**Figure 3:**
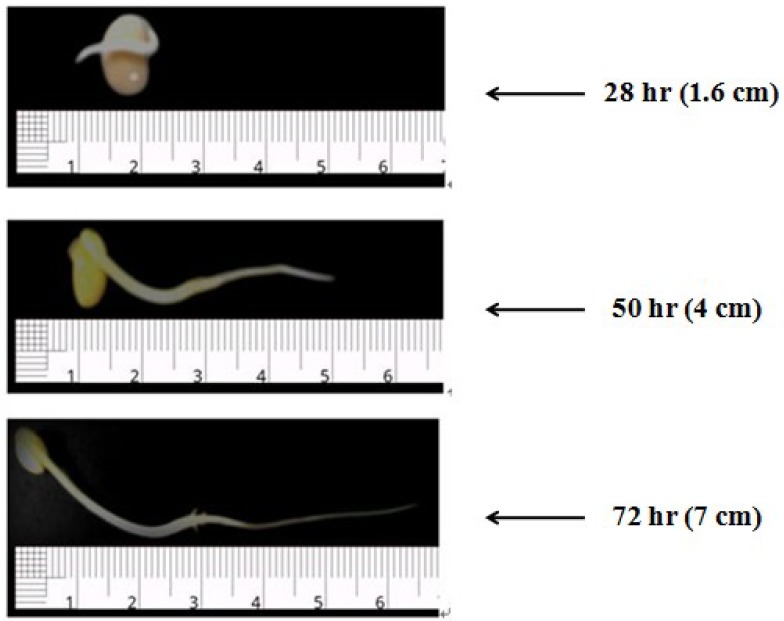
Radicle development of soybeans germinated for different times. The average length of radicles were sampled and given in parentheses.

It is well recognized that the germination effect of soybeans depends on several factors, including soaking time, humidity, germination time and temperature [6], and even under the same germination condition, different soybean cultivars may behave differently. Thus, these were all controlled at almost the same levels, which were also easily accessible and applicable, except germination time, being the only changing variable in the study condition. Figure 3 presents the radicles formed after 28 h, 50 h and 72 h of germination under the study condition. The small-seeded variety soybeans used in this study had a relatively higher germination rate as compared to common large-seeded varieties, and there was an abrupt increase in radicle growth after the initial 28 h. Generally, it takes four to six days for soybean sprouts to mature, but the germination time considered in this study is only confined to the first three days, since a shorter time will theoretically favor better retention of the original soy milk perception and minimize the production cost and labor caused by the additional germination step in soy milk manufacturing. Chauhan *et al.* [28] demonstrated that the sensory quality of soy beverages produced from germinated soybeans declined with increasing germination time, and two days was the limit without any significant changes in the sensory attributes. According to the present results, soy milk quality in terms of three classes of components, namely, total phenolic, phytic acid and trypsin inhibitors, was improved by the germination pre-treatment of soybeans carried out under the designed germination conditions. The minimal TIA was found in soy milk made with 28 h-germinated soybeans. Although the other two factors—TPC and phytic acid content—were both improved with the increase of germination time, the improvement was not significant (*p* < 0.05) after 28 h for TPC or after 50 h with respect to phytic acid. Thus, germination (1.6 cm sprout) at room temperature (~25 °C) for about 28 h was selected as the optimum germination condition for germinated soybean milk production, taking into account all aspects. Of course, the weight of different criteria for selecting the optimum condition can change with the purpose of product development.

### 3.5. Profile of Soy Milk Made from Germinated Soybean

The nutritional profile and some physicochemical properties of 28 h-germinated soybean milk are exhibited in Table 2 in relation to the non-germinated group. The pH of controlled soy milk is within the normal range (~ 6.7) determined by Liu and Chang [29], whereas it decreased a little in germinated soybean milk, possibly affected by the changed ash content. The small change is not supposed to greatly affect soy milk flavor, and salt ion concentration is purposed to have more influence rather than pH; however, the pH change may have some effect on the success of the process using soy milk produced to make other soy products (e.g., tofu), as it is demonstrated to be positively correlated with the coagulation of soy protein [29]. The proximate composition of controlled soy milk is comparable with Harjai and Singh’s investigations [30] in which soy milk contained about 6% total solids, 3% protein and 1.75% fat. The lower value in the present study most probably comes from the different water-to-bean ratio selected in soy milk production (8:1 in Harjai and Singh’s study), but may also rise from the cultivar difference of the soybeans used. After germination, total solid content dropped significantly (*p* < 0.05) in the produced soy milk, as a combined effect of the soybeans absorbing water and utilizing the constituents for germination and seedling growth [2]. Liu and Chang [7] found that total solids in soy milk might positively relate to carbohydrate content, which essentially correlates well with the results in the present study. Total carbohydrate content loss during germination is primarily the result of a decrease in simple sugars, low molecular weight oligosaccharides and starch, which are quickly consumed in the metabolic processes during germination [31]. This may improve the nutritional value of soy milk produced in terms of carbohydrate combination and eliminate flatulent problems related to soy milk consumption caused by raffinose and stachyose. Meanwhile, the higher moisture content making the soy milk diluted could be a reason contributing to the reduced viscosity. Though some tried to relate increment in viscosity to increase in protein content in soy milk [32], other research usually has inconsistent results [30]. Ash content also significantly reduced, which could result from leaching into the water, but some essential minerals, like Ca, Cu and Zn, could actually increase during the short-time germination, according to Kaushik *et al.* [3]. Fat content had reduced a little, but the change is not significant. On the other hand, protein content increased significantly in soy milk made from 28 h-germinated soybeans, which is reasonable, since germination of soybeans tends to increase their protein content [4,5] as a result of a net synthesis of enzyme proteins, especially during the early stage of germination, when hydrolysis of storage protein in cotyledons is minimum. Short-time germination is also shown to improve the protein quality in soybeans, as it promoted *in vitro* protein digestibility and generated a higher protein efficiency ratio [4] and may also benefit the overall protein quality of the soy milk produced. As protein is the most critical nutrient component in soy milk and daily consumption of 25 g of soy protein is recommended to reduce the risk of coronary heart disease, it is advantageous to increase protein concentration in soy milk. However, it is not clear in the present research whether the improved protein value is an effect of increased non-protein nitrogen, which was also observed to increase during soybean germination [31]. However, generally, changes with 28 h-germinated soybean milk composition provide comparable and even superior nutritional quality.

**Table 2:** Chemical and physical properties of soy milk made from non-germinated and germinated (28 h) soybean milk ^a^.

	Non-germinated soybean milk	Germinated soybean milk
Yield of milk (g of milk/100 g dry beans)	651.11 (0.96) A	619.44 (4.19) B
pH	6.72	6.51
Specific gravity	1.008 (0.006) A	1.002 (0.003) A
Viscosity (centipoises/cP)	3.46 (0.01) A	2.51 (0.06) B
Total solids (g/100 g liquid soy milk)	5.86 (0.39) A	4.76 (0.34) B
Moisture (g/100 g liquid soy milk)	94.14	95.24
Ash (g/100 g liquid soy milk)	0.33 (0.01) A	0.23 (0.01) B
Protein (g/100 g liquid soy milk)	2.19 (0.05) B	2.66 (0.02) A
Fat (g/100 g liquid soy milk)	1.37 (0.02) A	1.33 (0.04) A
Carbohydrate (g/100 g liquid soy milk)	1.96	0.54

^a^ Results are the means of triplicates, with standard deviation given in parentheses; Values marked with the same letter in the same row indicate no significant differences between two treatments (*p* < 0.05).

To better demonstrate the functional value, additional work is needed to make the germinated soybean milk suitable to be commercialized. For example, although the sensory attributes of the value-added soy milk produced in this project are not supposed to drastically deviate from traditional soy milk, it is necessary to set up a sensory panel to evaluate these attributes and to ensure acceptability by consumers; the stability of formulation is another factor to be considered. As mentioned earlier, germination, a single process, serves as a pool of possibilities for raw material improvement in soy milk production, since many of the improved attributes during soybean germination are valuable for soy milk. In the present study, only three classes of bioactive components were investigated, leaving behind possible improvements in other aspects, such as oligosaccharides (which cause flatulence), lipoxygenase activity (which cause beany flavor) and calcium content (an essential mineral). Analyses could be done to examine the level of these factors in the 28 h-germinated soybean milk, or other research could start from observing the effects of changing germination time on these factors. 

## 4. Conclusions

Extensive biochemical reactions take place during the process of soybean germination. Many of them lead to changes that are closely related to and can be key attributes in determining soy milk quality. In the present research, improvements in three classes of bioactive components investigated, namely, trypsin inhibitors, phytic acid and total phenolics, were observed simultaneously in cooked soy milk produced from short-time germinated soybeans. Optimum germination time was selected, and soy milk made from 28 h-germinated soybeans under optimum germination conditions showed significantly higher protein content, a lower amount of carbohydrate and a generally comparable physicochemical profile to traditional soy milk. The current approach seems to be a feasible and convenient way for the industry to develop functional soy milk products and provides practical data for industrial settings. Meanwhile, it offers a safer and more nutritious alternative for household soy milk consumers, who make their own soy milk at home. Besides, the findings in this study also imply that short-time germination could be a potential pre-treatment method to promote the functional properties in various soybean-derived food products.
